# Supplementary material for: Profiling the medical, functional, cognitive, and psychosocial care needs of adults assessed for home care in Ontario, Canada: The case for long-term ‘life care’ at home
Source: PLoS One. 2024 Apr 1;19(4):e0300521. doi: 10.1371/journal.pone.0300521 (PMC10984553; doi:10.1371/journal.pone.0300521)
Supplement: S1 Table — (PDF) [file pone.0300521.s001.pdf]

S1 Table. Plain Language Coding Rules for Service Groups Algorithm

Service Group	Variable(s) Used	Coding rules for inclusion in group
1 - Care coordination in conjunction with specialized geriatric services	i) 5Ms Geriatric Needs algorithm* (1)<br>ii) Changes in Health, End-Stage Disease, Signs and Symptoms (CHESS) Scale (2)<br>iii) Method for Assigning Priority Levels (MAPLe) (3)	a) Presence of four or more 5M indicators AND MAPLe is greater than or equal to 4 AND CHESS is greater than or equal to 3;<br>b) Otherwise go to step 2.
2 - Care coordination in conjunction with primary care	i) CHESS (2)<br>ii) MAPLe (3)<br>iii) Major comorbidity count‡ (4)	a) CHESS is greater than or equal to 3 AND MAPLe is greater than or equal to 4;<br>- OR -<br>b) CHESS is equal to 5;<br>- OR -<br>c) CHESS equals 3 or 4 AND one or more major comorbidity groups are present;<br>-OR -<br>d) Two or more major comorbidity groups are present;<br>e) Otherwise go to step 3.
3 - Care coordination for long-term home care with complex needs	i) CHESS (2)<br>ii) MAPLe (3)<br>iii) Caregiver distress<br>iv) Hours of informal care	a) CHESS equals 0, 1, or 2 AND MAPLe is greater than or equal to 4;<br>- OR -<br>b) Caregiver distress is present AND caregivers provide 14+ hours of care;<br>c) Otherwise go to step 4.
4 - Care coordination for long-term home care with moderate needs	i) Cognitive Performance Scale (CPS) (5)<br>ii) MAPLe (3)<br>iii) CHESS (2)	a) CPS is greater than or equal to 2 AND either: i) MAPLE=3 or ii) CHESS is greater than or equal to 3;<br>b) Otherwise go to step 5.
5 - Chronic Disease Management	i) CPS (5)<br>ii) MAPLe (3)<br>iii) CHESS (2)	a) CPS is less than 2 AND either: i) MAPLe equals 3 or ii) CHESS is greater than or equal to 3;<br>b) Otherwise go to step 6
6 - Monitor with self-reported interRAI Check Up assessment	NA	Default group if none of the above conditions are met.

* Considers indicators of impairment in Mind, Mobility, Medication, Multicomplexity, Matters Most
‡ Count of areas of comorbidity: heart, liver, lung, kidney, cancer, neurological conditions

References:

1. Hogeveen SE. Specialized Geriatric Services Use by Older Home Care Clients. Waterloo, Ontario: University of Waterloo; 2019.
2. Hirdes JP, Frijters DH, Teare GF. The MDS CHESS Scale A New Measure to Predict Mortality in Institutionalized Older People. Journal of the American Geriatrics Society. 2003;51(1):96-100.
3. Hirdes JP, Poss JW, Curtin-Telegdi N. The Method for Assigning Priority Levels (MAPLe): a new decision-support system for allocating home care resources. BMC Med. 2008;6:9.
4. Canadian Institute for Health Information. COVID-19 Major Comorbidity Count Algorithm for Long-Term Care Residents. Ottawa, ON; 2020.
5. Morris JN, Fries BE, Mehr DR, Hawes C, Phillips C, Mor V, et al. MDS Cognitive Performance Scale. Journal of Gerontology: MEDICAL SCIENCES. 1994;49(4):M174-M82.
