# Supplementary material for: Profiling the medical, functional, cognitive, and psychosocial care needs of adults assessed for home care in Ontario, Canada: The case for long-term ‘life care’ at home
Source: PLoS One. 2024 Apr 1;19(4):e0300521. doi: 10.1371/journal.pone.0300521 (PMC10984553; doi:10.1371/journal.pone.0300521)
Supplement: Attachment — Submitted filename: comment from reviewer.docx [file pone.0300521.s003.docx]

I think this paper needs minor revision to improve the rationale and gap of this study. Specific comment for each section is below:

**Introduction**

Page 4 Line 62: Please clarify why this study concerns only 4 domains? Why the domain related environment and social were excluded. Is it more effect in 4 domains in this population or there are some barrier to analysis the social and environmental domain?

**Methods**

Page 8 Line 149: How is the the population was grouped? Please explained the method of algorithm to select group

Page 12 Line 183: Please described the method for analysis Heat map of outcome measurement.

**Results**

Page 17 Table 5: A divergent color gradient is not related with the percent. It can make the reader confused. It should be have statement of adjusted the table to make it easier to understand and follow up on research results.

Page 19 Line 239: The figure 1 has not shown.

**Discussion**

Page 24 line 245: Please give some evidence to manage the barrier of time limited of . thrapists or interfrofessional focus on post-acute hospital

Page 24 line 353: In addition to social care, Caregiver education is also importance in psychological care and there are some results related to the caregiver. Please discuss on this topic

Page 25 line 373: Please show how to generalize the result of this study to improve the service before explaining the future study
